# Fe-Immobilised Catechol-Based Hypercrosslinked Polymer as Heterogeneous Fenton Catalyst for Degradation of Methylene Blue in Water

**DOI:** 10.3390/polym14132749

**Published:** 2022-07-05

**Authors:** Thanchanok Ratvijitvech

**Affiliations:** Department of Chemistry, Faculty of Science, Mahidol University, Bangkok 10400, Thailand; thanchanok.rat@mahidol.edu

**Keywords:** hypercrosslinked polymer, porous polymer, catechol, Fenton catalyst, dye degradation, methylene blue

## Abstract

Clean water is one of the sustainable development goals. Organic dye is one of the water pollutants affecting water quality. Hence, the conversion of dyes to safer species is crucial for water treatment. The Fenton reaction using Fe as a catalyst is a promising process. However, homogeneous catalysts are normally sensitive, difficult to separate, and burdensome to reuse. Therefore, a catechol-based hypercrosslinked polymer (catechol-HCP) was developed as an inexpensive solid support for Fe (catechol-HCP-Fe) and applied as a heterogenous Fenton catalyst. The good interaction of the catechol moiety with Fe, as well as the porous structure, simple preparation, low cost, and high stability of catechol-HCP, make it beneficial for Fe-loading in the polymer and Fenton reaction utilisation. The catechol-HCP-Fe demonstrated good catalytic activity for methylene blue (MB) degradation in a neutral pH. Complete decolouration of 100 ppm MB could be observed within 25 min. The rate of reaction was influenced by H_2_O_2_ concentration, polymer dose, MB concentration, pH, and temperature. The catechol-HCP-Fe could be reused for at least four cycles. The dominant reactive species of the reaction was considered to be singlet oxygen (^1^O_2_), and the plausible mechanism of the reaction was proposed.

## 1. Introduction

Clean water is one of the global sustainable development goals. The contamination by dyes in wastewater from industrial and domestic uses is one of the causes of water pollution [1]. Many dyes are non-biodegradable and toxic to both humans and aquatic life. Moreover, coloured water can prevent the penetration of light into bodies of water, leading to decreases in photosynthesis and oxygen in the water, which can affect the water ecosystem and further decrease water quality [2,3]. Therefore, the development of effective approaches to remove dyes from water is crucial.

Methods such as adsorption [4,5,6,7,8], coagulation [9,10,11,12,13,14], and membrane filtration [14,15,16,17,18] have been used for dye removal. However, such methods only transfer the pollutants from one place to another. Another interesting approach is the conversion of the dyes to safer species. The Fenton reaction is a promising process for degrading dyes into other species. The reaction involves the catalytic oxidation of organic compounds, including organic dyes, by using iron (Fe) and hydrogen peroxide (H_2_O_2_) as reagents [19,20,21,22,23]. Fenton catalysis has the advantages of being inexpensive, simple, and easy to use. Fe and H_2_O_2_ are inexpensive, and Fe is a highly abundant element on earth. Generally, the Fenton reaction can occur under UV or visible light [24,25,26,27]. Thus, no extra energy is required. Both homogeneous and heterogeneous catalysts can be applied in the Fenton reaction. Nonetheless, heterogeneous catalysts can overcome some limitations of homogeneous catalysts, such as the pH operating range and the stability of catalysts under the oxidation conditions. The undesired Fe sludge, which is normally generated in the homogeneous Fenton process, can be diminished by immobilising the Fe in heterogenous solid supports to prevent the leakage of the Fe into the system [28]. Moreover, solid catalysts are also easily separated from the reaction and have more potential for reuse. However, the reactivity of heterogeneous catalysts and the degradation of materials due to the harsh reaction conditions of the Fenton process are still challenging.

Hypercrosslinked polymers (HCPs) are amorphous polymeric materials with good stability [29,30]. HCPs typically have highly porous structures and large surface areas. HCPs with surface areas greater than 1000 m^2^/g can be obtained using benzene as a monomer [31]. However, the surface areas of HCPs vary depending on the monomers used and the structures of the HCPs formed. HCPs can be prepared using the Friedel–Crafts reaction, which is cheap and simple in comparison to many other methods. The pores and voids of HCPs can be designed to have the desired functionalities to enhance the interactions with guest molecules [32]. A wide range of monomers with different functional groups, e.g., hydrocarbons, halides, alcohols, and amines, can be used to prepare HCPs [31,33,34,35]. Moreover, HCPs also demonstrate high chemical and physical stabilities. Due to these properties, HCPs are utilised in many applications, such as gas capture and storage [36,37,38,39,40], pollutant adsorption [41,42,43,44], antibacterial [45], catalysis [46,47,48], and energy storage [49,50,51]. HCPs are considered to be potential candidates for heterogenous Fenton catalysis. The porous structure and interconnected pores of HCPs can promote the Fe-loading in the polymers, the accessibility of the active sites, and the diffusion of the guest molecules through the materials. Moreover, the porous structure of HCPs allows for the adsorption of dyes and acts in nanoconfinement to increase the concentration of the dyes in materials, which can enhance the degradation rate. The highly stable properties of HCPs are also beneficial in preventing the degradation of materials during the strong conditions of the Fenton reaction and in increasing the potential for reusing the materials so as to reduce material preparation costs. Porous organic polymers, the similar types of materials, based on ferrocene [52] and porphyrin [24] have been reported to have high catalytic efficiency. However, expensive chemicals and complex monomer and polymer preparations were employed. Due to the large scale of industrial waste, materials with low costs are required. Therefore, the development of materials using inexpensive starting materials and simple preparation methods, which can preserve catalytic efficiency, is needed.

The expense of materials can be defined by their abundance, preparation process, efficiency, and reusability [53]. Catechol is an inexpensive and abundant compound widely found in nature and used in industries. The catechol structure has also been found to interact well with Fe. Recently, our group reported the enhancement of Fe adsorption in HCP using the catechol moiety [54]. Apart from increasing the interaction with Fe, the catechol moiety can also behave as a chelating agent to increase the regeneration of Fe^2+^ in the material, which can increase the catalytic activity of the Fenton reaction [55,56]. Thus, herein, the HCP synthesised by catechol monomer, catechol-based HCP (catechol-HCP), using inexpensive and simple chemicals and methods, was utilised as a solid support for immobilising Fe in Fenton dye-degradation catalysis. Methylene blue was used as a dye model. The Fe-immobilised catechol-HCP (catechol-HCP-Fe) was investigated for its efficiency in the degradation of MB. The catechol-HCP-Fe demonstrated good catalytic activity in MB degradation. Factors, including H_2_O_2_ concentration, polymer dose, initial MB concentration, pH, and reaction temperature, were found to influence the rate of reaction. The reusability of the material was also studied. The key reactive species and the plausible mechanism of the reaction were also investigated.

## 2. Materials and Methods

### 2.1. Chemicals and Reagents

Catechol was obtained from Acros Organics (Geel, Belgium). Formaldehyde dimethyl acetal (FDA), ferrous ammonium sulphate hexahydrate (Fe(NH_4_)_2_(SO_4_)_2_·6H_2_O), and hydrogen peroxide (H_2_O_2_) were purchased from Merck (Darmstadt, Germany). Iron (III) chloride (FeCl_3_), 1,2-dichloroethane (DCE), sodium acetate anhydrous, and sodium hydroxide (NaOH) were purchased from Carlo Erba Reagents (Paris, France). Methylene blue (MB), ethylenediaminetetraacetic acid disodium salt (EDTA·2Na), 1,10-phenanthroline (*o*-phen), and hydroxylamine hydrochloride were purchased from Ajax Finechem (New South Wales, Australia). *Tert*-butanol (TBA), *p*-benzoquinone (*p*-BQ), and L-histidine (L-His) were purchased from TCI (Tokyo, Japan). Hydrochloric acid (HCl, 37%) was purchased from RCI Labscan (Bangkok, Thailand). All commercially available chemicals and solvents were used as received.

### 2.2. Characterisation Apparatus

Fourier transform infrared (FT-IR) spectra were recorded from a Frontier FT-IR Spectrometer (PerkinElmer, Waltham, MA, USA), using KBr disks. CHN elemental analysis was performed using a CHNS/O Analyser (Thermo Scientific^TM^ FLASH 2000, Thermo Fisher Scientific, Waltham, MA, USA). Material surface morphology images and elemental analysis were collected by a field emission scanning electron microscope (FESEM, SU-8010, Hitachi, Tokyo, Japan) equipped with an EDAX Element Energy Dispersive Spectroscopy System. Thermogravimetric analysis (TGA) was conducted via SDT 2960 DSC-TGA (TA Instruments, New Castle, DE, USA) in an air atmosphere by heating to 800 °C at a rate of 10 °C/min. Nitrogen isotherms were collected on a 3Flex gas sorption analyser (Micromeritics, Norcross, GA, USA) at 77 K. Samples were degassed at 120 °C overnight. Surface areas were calculated using the Brunauer–Emmett–Teller (BET) theory in the relative pressure range of 0.05–0.5. UV–Visible spectra were recorded by a UV-1800 UV–Vis spectrophotometer (Shimadzu, Tokyo, Japan).

### 2.3. Preparation of Fe-Immobilised HCP (Catechol-HCP-Fe)

Catechol (0.1 mol, 11 g) in 1,2-dichloroethane (100 mL) was added to formaldehyde dimethyl acetal (FDA) (0.2 mol, 18 mL) and iron (III) chloride (FeCl_3_) (0.2 mol, 32 g). After heating at 80 °C for 24 h, the reaction was filtered. The solid was washed with methanol until the filtrate was colourless and then dried in an oven at 80 °C overnight. An insoluble black powder of catechol-HCP was obtained (13 g, 99%).

Before the Fe-immobilisation process, the catechol-HCP was washed with 1 M HCl solution to remove the residual Fe from the synthetic process. The polymer was repeatedly washed until less than 1 ppm of Fe leaching into the solution was observed. The polymer was further washed with distilled water until the filtrate was neutral. After that, the washed polymer was dried in an oven at 80 °C overnight to obtain catechol-HCP-w (12 g, 94%); elemental analysis: C 62.68%, H 3.56%.

After the catechol-HCP was washed, Fe was loaded into the polymer by immersing the prepared polymer (1 g) in the Fe^2+^ solution (500 ppm, 100 mL). The solution was stirred at room temperature for 24 h and then filtered to obtain catechol-HCP-Fe as a black powder (1 g, 97%); elemental analysis: C 58.83%, H 3.44%.

The catechol-HCP and catechol-HCP-Fe were characterised by Fourier transform infrared (FT-IR) spectroscopy, elemental analysis, scanning electron microscopy (SEM), energy dispersive X-ray analysis (EDX), thermogravimetric analysis (TGA), and surface area analysis.

### 2.4. Fe-Loading Determination in Catechol-HCP-Fe

The amount of Fe loading in the catechol-HCP-Fe was calculated by the difference of the Fe concentration in the solution, before and after filtration. The concentration of Fe in the solution was determined by the colourimetry technique. The Fe in the solution was complexed with 1,10-phenanthroline (*o*-phen) to form the colour complex. Hydroxylamine hydrochloride and sodium acetate buffer pH 5 were used as a reducing agent and a pH-adjusting solution, respectively. An UV-Visible spectrophotometer was used to measure the absorbance of the solutions at 510 nm related to the absorption maximum wavelength of the Fe complex. The Fe loading in the catechol-HCP-Fe was calculated as being around 25 mg/g.

### 2.5. Dye Degradation Experiments

The Fenton catalytic activity of the materials was evaluated by their methylene blue (MB) degradation efficiency. A known concentration of dye solution (50 mL) was added to the prepared polymer. The mixture was added to a hydrogen peroxide (H_2_O_2_) solution and stirred (500 rpm) at room temperature. The typical experiments were done at a neutral pH (pH = 6) using deionised water. Due to the fact that the pH of deionised water is slightly lower than that of normal water (pH = 7), a pH of 6 was considered as a neutral pH in this work. The pH was further adjusted to obtain a pH range of 3–11 by using 1 M HCl and 1 M NaOH. The reaction solution was collected at a specific time so as to study the reaction kinetics. The MB concentration was determined by an UV-Visible spectrophotometer at 664 nm. The percentage of degradation was calculated using Equation (1):

Degradation (%) = (*C*_0_ − *C_t_*) × 100/*C*_0_ (1)

where *C*_0_ and *C_t_* are the concentrations of MB at 0 and *t* min, respectively.

For the reuse experiments, the catechol-HCP-Fe was filtered from the reaction, washed with water several times, and dried in an oven at 80 °C for 24 h. The material was then reused for the next cycle.

For the mechanistic study, 1 mM of scavenger was added to the reaction mixture using a 1 g/L catechol-HCP-Fe, 0.25 M H_2_O_2_, 50 mL (100 ppm) MB solution, pH 6 (neutral pH), which was stirred (500 rpm) at room temperature. *Tert*-butanol (TBA), *p*-benzoquinone (*p*-BQ), L-histidine (L-His), and ethylenediaminetetraacetic acid disodium salt (EDTA) were used as the ·OH, ·O_2_^−^, ^1^O_2_, and photo-generated h^+^ scavengers, respectively.

## 3. Results and Discussion

### 3.1. Preparation and Characterisation of Fe-Immobilised HCP (Catechol-HCP-Fe)

#### 3.1.1. Preparation of Fe-Immobilised HCP (Catechol-HCP-Fe)

**Scheme 1:**
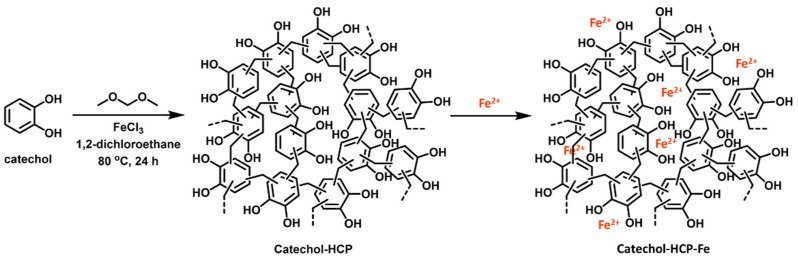
Preparation of Catechol-HCP-Fe.

Fe-immobilised HCP (catechol-HCP-Fe) was prepared via the synthesis of catechol-based HCP (catechol-HCP), followed by the immobilisation of Fe in the catechol-HCP (Scheme 1).

Catechol-HCP was synthesised by extensive crosslinking of the catechol monomer with an external crosslinker, formaldehyde dimethyl acetal (FDA), using FeCl_3_ catalyst via a Friedel–Crafts alkylation reaction. A black powder of catechol-HCP was obtained in a good yield (99%). The result also demonstrated the possibility of scaling up the synthesis of material as a 10-g-scale of material was successfully synthesised without the reduction of the yield as compared to the smaller-scale synthesis reported in the literature [54].

Before the immobilisation process, the synthesised catechol-HCP was washed with HCl to remove the residual Fe from the synthetic process. To immobilise the Fe in the material, the washed catechol-HCP (catechol-HCP-w) was immersed in the Fe solution for 24 h and filtered to obtain the catechol-HCP-Fe. The amount of Fe adsorbed in the polymer was calculated by the difference of the Fe concentration in the solution, before and after filtration. By using 100 mL of 500 ppm Fe^2+^ solution and 1 g of catechol-HCP, around 250 ppm was found adsorbed in the polymer, calculated as being approximately 25 mg/g Fe-loading in the material.

#### 3.1.2. Fourier Transform Infrared (FT-IR) Spectroscopy

**Figure 1:**
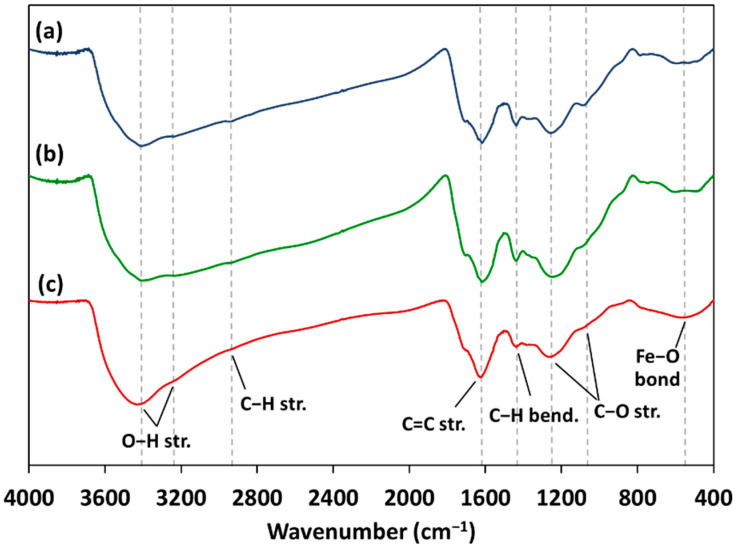
IR spectra of (**a**) catechol-HCP, (**b**) catechol-HCP-w, and (**c**) catechol-HCP-Fe.

Chemical structures of the prepared polymers were characterised by IR spectroscopy (Figure 1). IR spectra of catechol-HCP (as synthesised), catechol-HCP-w (after washed), and catechol-HCP-Fe (after Fe immobilisation) illustrated similar peaks, indicating that the chemical structure of the material did not significantly change during the modification process. Aromatic C=C stretching peaks around 1600 cm^−1^ and C-H stretching and bending peaks around 2900 and 1440 cm^−1^ demonstrated the aromatic backbone of the catechol moiety and the methylene group of the crosslinker, respectively. A broad O-H stretching peak around 3200 cm^−1^ and C-O stretching peaks around 1090 cm^−1^ indicated the presence of alcohol (-OH) groups in the materials. Other O-H and C-O stretching peaks observed at around 3400 and 1250 cm^−1^ could be attributed to the moisture, which might be adsorbed in the porous structure of the material, and the ether functional group, which might remain after the incomplete crosslinking reaction, respectively. Nevertheless, the intensity of the peak around 570 cm^−1^, corresponding to Fe-O bonds, slightly decreased after the material was washed, and it increased again after the Fe was immobilised, suggesting the success of Fe removal and incorporation in the material [57,58].

#### 3.1.3. Elemental Analysis

Elemental contents in the materials were analysed by CHN elemental analysis. The results are demonstrated in Table 1. C and H contents lower than the theoretical values were typically found in the HCPs, which could possibly be due to the adsorption ability of the materials in adsorbing molecules and moisture from the air and the higher-than-expected O content in the HCPs, resulting from the incomplete crosslinking reaction. However, comparing the catechol-HCP-w and the catechol-HCP-Fe, decreases in C and H contents in the polymer after Fe immobilisation were observed as expected due to the incorporation of Fe in the polymer.

#### 3.1.4. Scanning Electron Microscopy (SEM) and Energy Dispersive X-ray (EDX) Analysis

**Figure 2:**
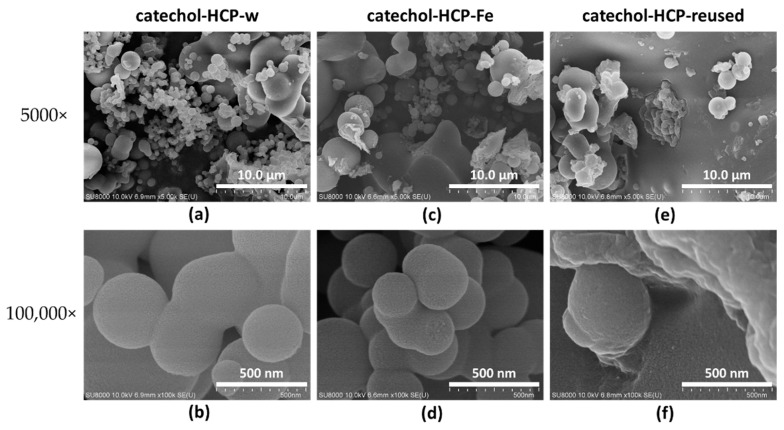
SEM images of catechol-HCP-w at (**a**) 5000× and (**b**) 100,000×; catechol-HCP-Fe at (**c**) 5000× and (**d**) 100,000×; and catechol-HCP-reused at (**e**) 5000× and (**f**) 100,000×.

The surface morphology of the materials was perceived by a scanning electron microscope. Figure 2 illustrates the SEM images of the catechol-HCP-w (a,b) and the catechol-HCP-Fe (c,d). The polymer, after four reuse cycles, catechol-HCP-reused, was also investigated as demonstrated in Figure 2e,f. The surface morphology of the materials did not significantly change after the Fe immobilisation and reuse steps, implying the endurance of the materials under the operating conditions. While the EDX spectrum of the catechol-HCP-w (Figure S1 from the Supplementary Materials) did not show Fe peaks, the peaks appeared in the spectrum of catechol-HCP-Fe (Figure S2) after Fe immobilisation. As predicted, the EDX elemental analysis (Table S1) also elucidated a decrease in the carbon ratio and an increase in the Fe ratio in the catechol-HCP-Fe as compared to those in the catechol-HCP-w.

#### 3.1.5. Thermogravimetric Analysis (TGA)

The thermal stability of the prepared materials was analysed using the TGA technique. The TGA spectra of catechol-HCP-w and catechol-HCP-Fe are shown in Figures S3 and S4, respectively. The weight loss around 100 °C resulted from the loss of water moisture adsorbed in the polymers. The materials, both with and without Fe, were thermally stable with heating up to around 250 °C in an air atmosphere. The decomposition of materials took place at around 250–600 °C. Upon reaching 800 °C, the catechol-HCP-w produced char around 0.5%, while around 1.8% of char was obtained in the case of the catechol-HCP-Fe. The higher amount of char left, which could be from the unburned Fe, also pointed to the incorporation of Fe in the catechol-HCP-Fe.

#### 3.1.6. Surface Area Analysis

**Figure 3:**
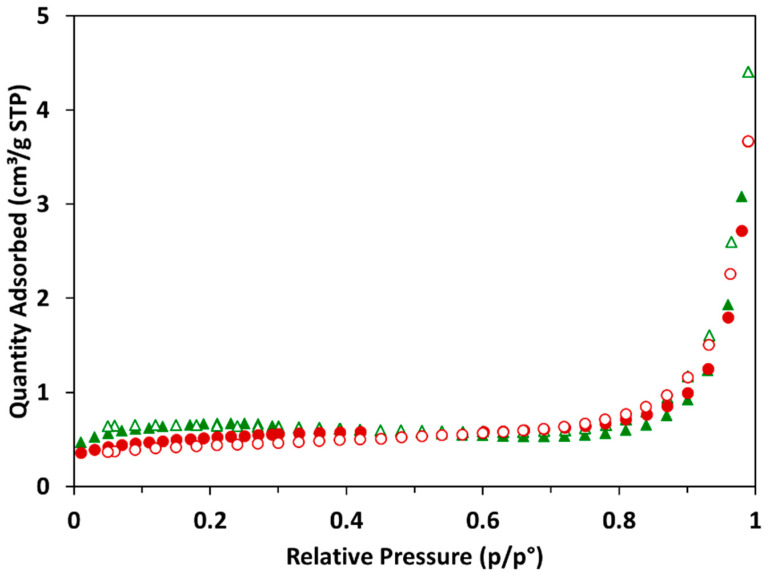
Nitrogen isotherms of catechol-HCP-w (green triangle) and catechol-HCP-Fe (red circle). The closed and open symbols represent the adsorption and desorption isotherms, respectively.

The porosity of the materials was analysed by nitrogen sorption isotherms at 77 K (Figure 3). The isotherms of the catechol-HCP before (catechol-HCP-w, green triangle) and after (catechol-HCP-Fe, red circle) Fe immobilisation demonstrated the similar shapes and gas sorption capabilities. The surface areas, calculated by the Brunauer–Emmet–Teller (BET) theory, of the catechol-HCP-w and catechol-HCP-Fe were around 2.4 and 1.9, respectively, comparable to the reported values [54]. After the Fe was immobilised, a slight decline in surface area was observed, implying the adsorption of Fe into the porous structure. The pore volume of the material also diminished after Fe immobilisation. The total pore volume declined slightly, from 2.57 to 2.46 mm^3^/g, while the micropore volume declined more significantly, from 0.78 to 0.32 mm^3^/g, suggesting that the Fe was better-adsorbed into the microporous structure of the material. Despite the low measured surface area, the material showed a good Fe-adsorption capacity. This could imply the existence of the porous structure of the material. The low value of surface areas could possibly be due to many reasons. The -OH substituents on the catechol monomer could have formed H-bonding or have filled the pores, leading to the lower surface areas [59]. Moreover, the pores might collapse in the dry state. However, HCPs were found to be swellable when adsorbing the solvents or guest molecules [37,60]. Thus, although the low surface areas were observed, the material is still considered as the good adsorbent.

### 3.2. Catalytic Property of Catechol-HCP-Fe

The Fenton catalytic property of the prepared catechol-HCP-Fe was examined by adding catechol-HCP-Fe into the MB solution before adding H_2_O_2_ to the reaction mixture. For comparison, experiments using catechol-HCP-Fe mixed with MB without H_2_O_2_, MB solution with H_2_O_2_, and MB solution were conducted to confirm that the dye was not simply adsorbed into the polymer, degraded with H_2_O_2_, or self-degraded in the experimental environment, respectively.

**Figure 4:**
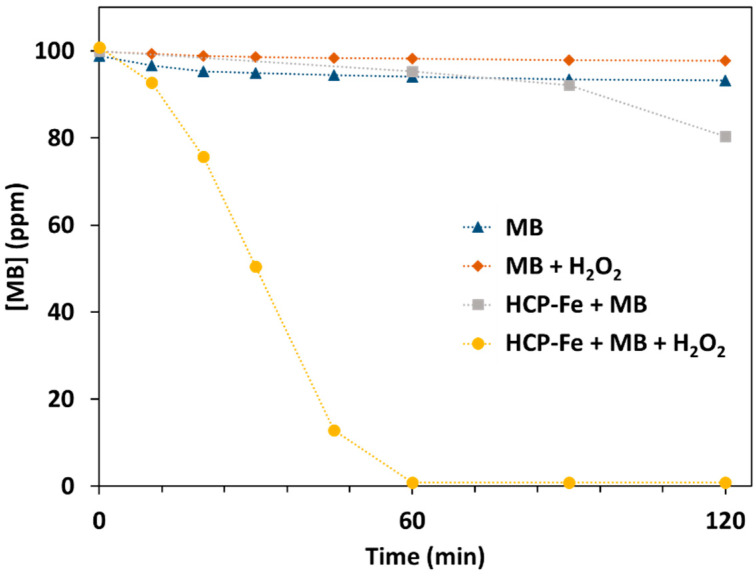
Concentrations of MB at different times under different conditions. Reaction conditions: [HCP-Fe] = 1 g/L, [H_2_O_2_] = 0.5 M, [MB] = 100 ppm, pH = 6 (neutral pH), room temperature (27 °C).

The results are shown in Figure 4. The concentrations of the MB in the MB solution (MB) and the MB mixed with H_2_O_2_ solution (MB + H_2_O_2_) were only slightly changed, indicating that the self-degradation of MB, both with and without H_2_O_2_, occurred only minimally in the experimental environment. The adsorption of the MB by the catechol-HCP-Fe was observed, as shown by the slight decrease in the MB concentration when the catechol-HCP-Fe was added to the solution without H_2_O_2_ (HCP-Fe + MB). The ability of the HCP in adsorbing MB would be beneficial in the mass transportation of the guest molecules to the active sites of the material. However, compared to the experiment with the catechol-HCP-Fe in the presence of H_2_O_2_ (HCP-Fe + MB + H_2_O_2_), which demonstrated a significant decrease in the MB concentration, the adsorption process was much slower than the catalytic process. This could confirm the catalytic ability of the catechol-HCP-Fe in the degradation of the MB.

To accurately calculate the kinetics of the MB degradation reaction, the data were fitted with zero-order, first-order, and second-order kinetic models using Equations (2)–(4), respectively:

*C_t_* = −*k*_0_*t* + *C*_0_ (2)

*ln C_t_* = −*k*_1_*t* + *ln C*_0_ (3)

1/*C_t_* = *k*_2_*t* + 1/*C*_0_ (4)

where *C*_0_ and *C_t_* are the concentrations of the MB at 0 and *t* min, and *k*_0_, *k*_1_, and *k*_2_ are zero-order, first-order, and second-order rate constants, respectively. Considering the curve-fitting (Figure S5) and *R*^2^ values (Table 2), the zero-order kinetic model demonstrated the best fit to the experimental data.

It is worth noting that most of the previously reported kinetic results followed the first-order kinetic model, where the reaction was slower with the time of reaction. This could be caused by the accumulation of intermediates in the material, which could hinder the active sites, leading to the lower reaction rate [52,58,61]. In comparison, the zero-order kinetics in this work suggested that the reaction was not slower with the reaction time and the porous structure of the HCP could promote mass transportation in the material. A similar result was also observed in the porphyrin-based porous material [24].

### 3.3. Effect of Parameters on MB Degradation

To optimise the catalytic efficiency of the catechol-HCP-Fe in the dye-degradation process, parameters that could affect the catalytic activity, including the H_2_O_2_ concentration, catechol-HCP-Fe dose, initial dye concentration, pH, and temperature, were studied.

#### 3.3.1. Effect of H_2_O_2_ Concentration

**Figure 5:**
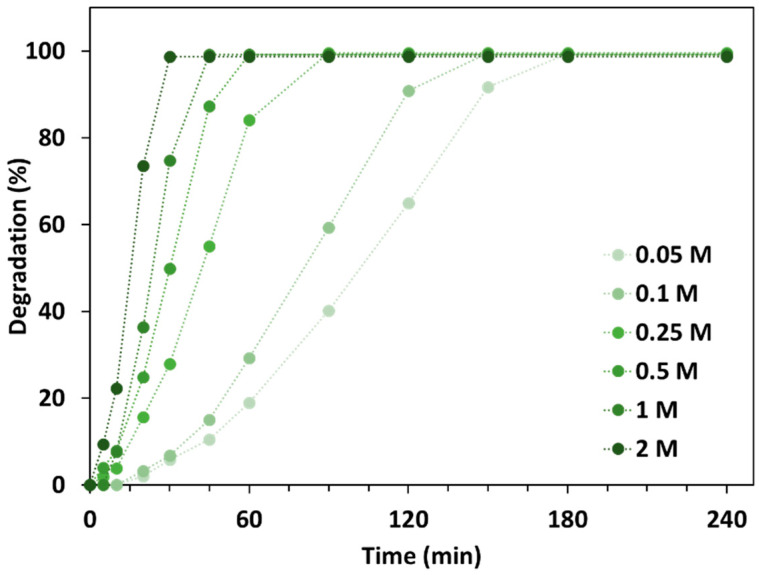
Degradation efficiency using different H_2_O_2_ concentrations. Reaction conditions: [catechol-HCP-Fe] = 1 g/L, [MB] = 100 ppm, pH = 6 (neutral pH), room temperature (27 °C).

**Figure 6:**
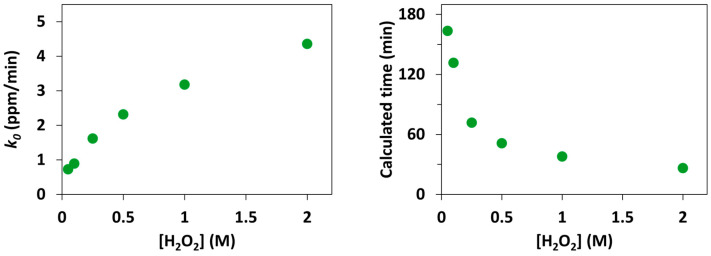
Rate constant (**left**) and calculated time (**right**) of MB degradation using different H_2_O_2_ concentrations.

To investigate the effect of the H_2_O_2_ concentration on the catalytic efficiency, different concentration of H_2_O_2_ (0.05, 0.1, 0.25, 0.5, 1, and 2 M) were added to the reactions. The concentrations were the final concentrations of H_2_O_2_ in the reaction mixtures. The dye-degradation efficiency, using various H_2_O_2_ concentrations, is demonstrated in Figure 5. The rate of reaction increased with the increase in the H_2_O_2_ concentrations, as shown in Figure 6 (left). The estimated reaction time of the reaction, illustrated in Figure 6 (right), was also calculated. The dye degradation occurred faster with a higher concentration of H_2_O_2_. The reaction time was reduced from more than 160 min to less than 30 min when the H_2_O_2_ concentration was increased from 0.05 to 2 M. However, the reaction time significantly decreased when the H_2_O_2_ concentration was increased from 0.05 to 0.5 M, but increased slightly when the H_2_O_2_ concentration was further increased to 1 and to 2 M. Therefore, considering the efficiency of the reagent-spending, a H_2_O_2_ concentration of 0.5 M was used in further experiments.

#### 3.3.2. Effect of Polymer Dose

**Figure 7:**
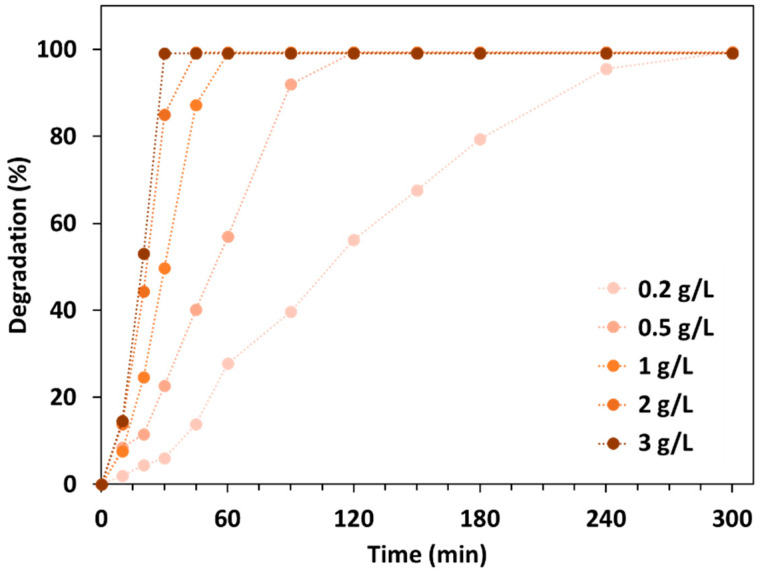
Degradation efficiency using different catechol-HCP-Fe doses. Reaction conditions: [H_2_O_2_] = 0.5 M, [MB] = 100 ppm, pH = 6 (neutral pH), room temperature (27 °C).

**Figure 8:**
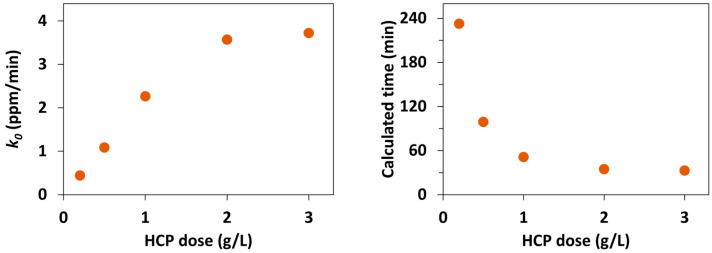
Rate constant (**left**) and calculated time (**right**) of MB degradation using different catechol-HCP-Fe doses.

The amount of catalyst could also influence the rate of the catalytic reaction. Therefore, the amount of the catechol-HCP-Fe was varied from 0.2 to 3 g/L. The results are illustrated in Figure 7. The dye degradation occurred faster with increases in the catechol-HCP-Fe dose. The rate of reaction could be accelerated by using a higher dose of the catalyst. The plateau was reached at 2 g/L of catechol-HCP-Fe, as demonstrated in Figure 8 (left). More than 99% of 100 ppm MB could be degraded in around 30 min using 2 g/L of catechol-HCP-Fe and 0.5 M H_2_O_2_. However, considering the time efficiency, (see Figure 8 (right)), the reaction time significantly decreased when the dose of catechol-HCP-Fe was increased from 0.2 to 1 g/L and levelled off when the dose was further increased to 2 and to 3 g/L. Thus, a catechol-HCP-Fe dose of 1 g/L was used in further investigations. An experiment using 2 g/L of catechol-HCP-Fe and 2 M of H_2_O_2_ was also carried out to optimise the reaction time; the complete degradation of the MB was observed within 25 min.

#### 3.3.3. Effect of Initial Dye Concentration

**Figure 9:**
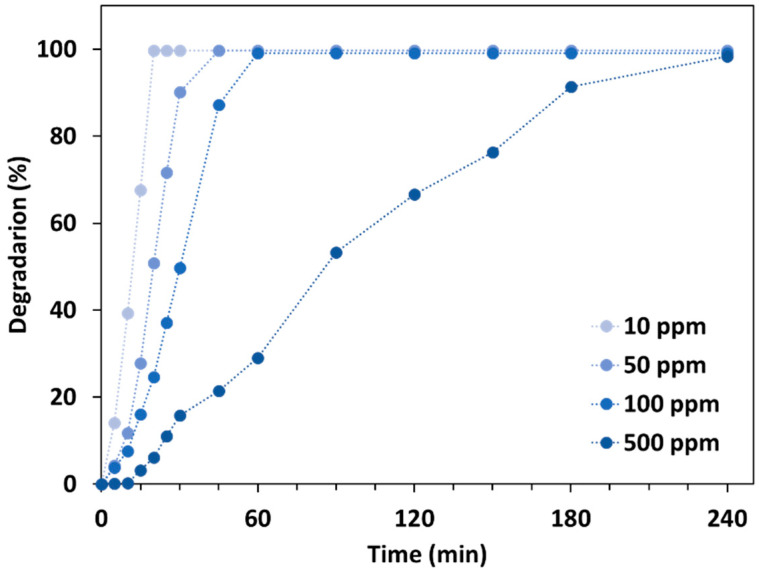
Degradation efficiency using different initial MB concentrations. Reaction conditions: [catechol-HCP-Fe] = 1 g/L, [H_2_O_2_] = 0.5 M, pH = 6 (neutral pH), room temperature (27 °C).

**Figure 10:**
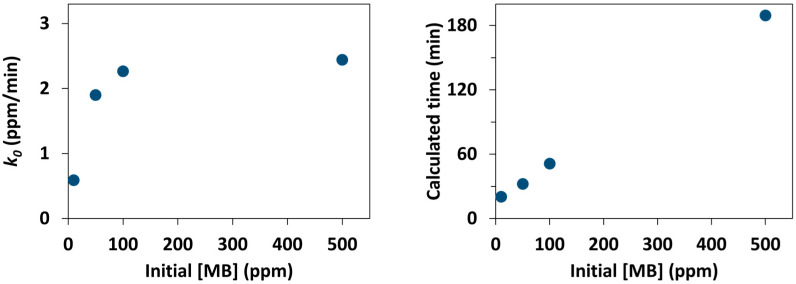
Rate constant (**left**) and calculated time (**right**) of MB degradation using different initial MB concentrations.

Another factor that could affect the dye-degradation efficiency is the initial dye concentration. Initial MB concentrations of 10, 50, 100, and 500 ppm were compared. The dye-degradation efficiency, the rate constant, and the estimated time of the reaction are shown in Figure 9 and Figure 10. With an increase in the initial MB concentration, a longer time for the dye degradation was required, owing to a larger number of dye molecules needing to be transformed while a limited number of active sites were available at one time. However, the rate constant increased with the increase in initial dye concentration as a greater number of molecules were residing near the active sites, along with the good mass transportation in the material, leading to a faster rate of reaction. Interestingly, the time of the reaction seemed to be linearly correlated to the initial dye concentration used in the reaction. Thus, the reaction time could be estimated if the initial MB concentration were known. Moreover, water contaminated with MB in amounts of up to 500 ppm could be treated by catechol-HCP-Fe within a couple of hours.

#### 3.3.4. Effect of Initial pH of Dye Solution and Reaction Temperature

**Figure 11:**
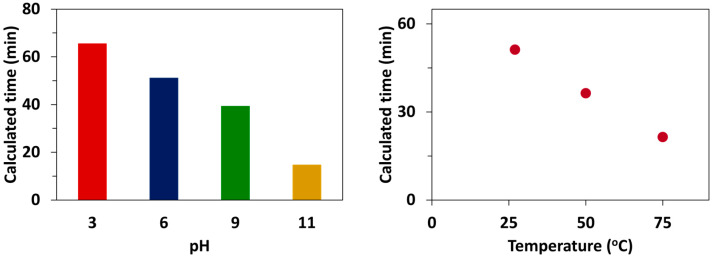
Calculated time of MB degradation using different pH levels (**left**) and temperatures (**right**). Reaction conditions: [catechol-HCP-Fe] = 1 g/L, [H_2_O_2_] = 0.5 M, [MB] = 100 ppm, pH = 6 (neutral pH) or as specified, room temperature (27 °C) or as specified.

The pH and temperature of wastewater might be unpredictable. Therefore, the effects of these factors on the Fenton-reaction efficiency were studied. Nearly 100% of 100 ppm of MB could be degraded by the catechol-HCP-Fe within a pH range of 3 to 11 (Figure S6) and a reaction temperature range of around 27 °C (room temperature) to 75 °C (Figure S7). Many have reported an optimum pH of around 3 for the Fenton process [62,63,64]. Therefore, the extra step and reagents to acidify the system were needed. However, our catalyst demonstrated good catalytic efficiency in a wide pH range. Thus, the extra cost and step of adjusting the pH could be omitted. More surprisingly, unlike traditional Fenton reactions where the optimum pH is in acidic range, the catechol-HCP-Fe demonstrated a slightly slower reaction rate in acidic conditions and a faster reaction rate in basic conditions, as shown in Figure 11. The reaction temperature also affected the rate of MB degradation. As expected, increasing the reaction temperature accelerated the reaction rate (Figure 11) due to the increase in kinetic energy in the system.

The experiments showed that the MB-degradation efficiency of the catechol-HCP-Fe was affected by the reaction conditions. The degradation rate increased with increases in the H_2_O_2_ concentration, catalyst dose, and initial dye concentration. The optimum condition concerning reagent spending was 1 g/L catechol-HCP-Fe and 0.5 M H_2_O_2_, in which 100 ppm of MB could be degraded in around 50 min at room temperature and with a neutral pH. However, an optimum time of around 23 min was obtained using 2 g/L catechol-HCP-Fe and 2 M H_2_O_2_ for 100 ppm of MB at room temperature and with a neutral pH. The catechol-HCP-Fe was also proven to be efficiently used in a wide operating range, pH of 3–11 and temperature of 27–75 °C. Compared with the reported materials (Table 3), catechol-HCP-Fe is considered to be an inexpensive material with a good catalytic property, and thus having a potential use in industrial wastewater management.

### 3.4. Reusability of Catechol-HCP-Fe for Dye Degradation

**Figure 12:**
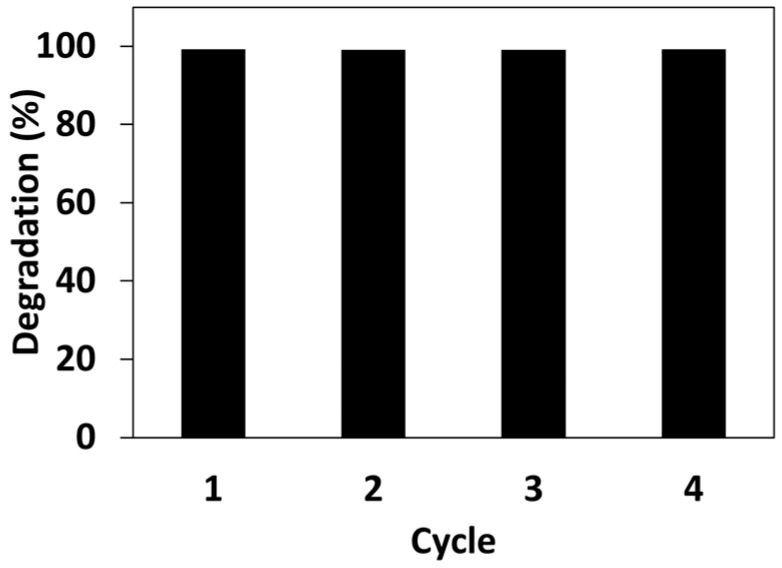
Degradation efficiency of the reused catalyst. Reaction conditions: [catechol-HCP-Fe] = 1 g/L, [H_2_O_2_] = 0.5 M, [MB] = 100 ppm, pH = 6 (neutral pH), room temperature (27 °C).

To evaluate the stability and reusability of the catalyst, the catechol-HCP-Fe was filtered from the solution after the reaction was done, washed several times with water, and dried in an oven before reuse. More than 99% of the MB could be degraded using the reused catalyst for at least 4 cycles (Figure 12). However, the rate of reaction dropped when the catalyst was reused, and a longer time was required for complete degradation (Figure S8). This could be responsible from the leaching of Fe into the solution during the reaction, causing less Fe to remain in the catalyst. However, only 0.5–2 mg/g of the Fe was found in the solution, calculated as only 2–8% Fe leaching, as compared to the amount of Fe loaded in the polymer. To improve the rate of the reaction, the Fe was reloaded into the reused catalyst prior to the new reuse cycle. By using the catalyst with reloaded Fe, it was found that the reaction time was comparable to the freshly prepared catalyst, as shown in Figure S9. These results could demonstrate that catechol-HCP-Fe can be reused and regenerated for catalytic dye-degradation reactions.

### 3.5. Mechanistic Study of Dye Degradation Process

**Figure 13:**
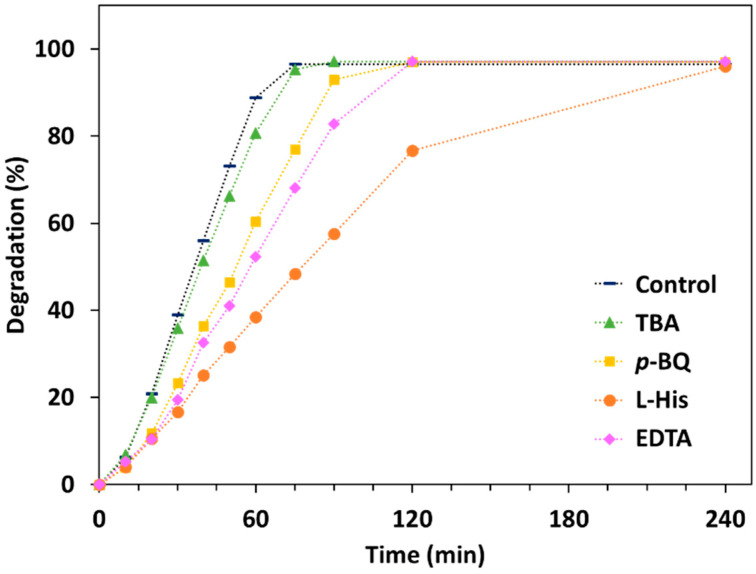
Effect of scavengers on the MB-degradation efficiency of catechol-HCP-Fe. Reaction conditions: [scavenger] = 1 mM, [catechol-HCP-Fe] = 1 g/L, [H_2_O_2_] = 0.5 M, [MB] = 100 ppm, pH = 6 (neutral pH), room temperature (27 °C).

The catalytic mechanism of the catechol-HCP-Fe was also studied by using trapping reagents. The major active species in the Fenton catalytic process are believed to be ·OH, ·O_2_^−^, and ^1^O_2_. Thus, *tert*-butanol (TBA), the ·OH scavenger, *p*-benzoquinone (*p*-BQ), the ·O_2_^−^ scavenger, L-histidine (L-His), the ^1^O_2_ scavenger, and ethylenediaminetetraacetic acid disodium salt (EDTA), the photo-generated h^+^ scavenger, were added to the reaction mixtures. As demonstrated in the results shown in Figure 13, the rate of the reaction decreased when the scavengers were added to the reaction, indicating the responsibility of the active species in the Fenton catalytic process using catechol-HCP-Fe. Interestingly, the trapping of ·OH, the typical prominent species in Fenton catalysis, slightly affected the reaction rate, suggesting that the ·OH might not be the main active species in this reaction mechanism. Instead, the dominant active species was considered to be ^1^O_2_, owing to the significantly longer reaction time when L-His was added to the reaction. The ·O_2_^−^ and photo-generated h^+^ were also involved in the reaction mechanism.

According to the experimental results, the postulated reaction mechanism for the MB degradation of catechol-HCP-Fe could be as follows:

Fe^3+^ + H_2_O_2_ → Fe^2+^ + ·O_2_^−^ + 2H^+^ (5)

2 ·O_2_^−^ + 2H_2_O → 2 ^1^O_2_ + H_2_O_2_ + 2H^+^ (6)

Fe^2+^ + 2 ·O_2_^−^ + 4H^+^ → Fe^3+^ + ^1^O_2_ + 2H_2_O (7)

MB + ^1^O_2_ → CO_2_ + H_2_O + other products (8)

It is worth noting that, even though ·OH has commonly been found to be the main active species for Fenton reactions [52,58,70], some research has also reported ^1^O_2_ to be the key species [24,61,71]. The nanoconfinement of the Fe in the porous structure of catechol-HCP-Fe might stimulate the generation of ^1^O_2_, as Yang et al. demonstrated that ^1^O_2_ was the preferably predominant species in the nanoconfined structure of Fe_2_O_3_@FCNT, while the ·OH was the main active species when the Fe_2_O_3_ was anchored on the outer surface of the FCNT [61]. The catechol moiety was also found to induce ^1^O_2_ production, as reported by Bokare and Choi [72]. This diverse mechanism could extend the opportunities for employing the material in broader reaction conditions and utilisations.

## 4. Conclusions

The Fe-immobilised catechol-HCP (catechol-HCP-Fe) was successfully prepared by the synthesis of catechol-HCP, followed by the immobilisation of Fe in the material. The large-scale synthesis of catechol-HCP could provide good yields, and the catechol-HCP was fully characterised by FT-IR spectroscopy, CHN elemental analysis, SEM, EDX, TGA, and surface area analysis. The amount of Fe immobilised in the catechol-HCP-Fe was approximately 25 mg/g.

The prepared material was studied for its catalytic activity in a Fenton reaction for the degradation of methylene blue (MB), the dye model. The parameters, including H_2_O_2_ concentration, catechol-HCP-Fe dose, initial dye concentration, pH, and temperature, were found to affect the reaction rate. Increasing the H_2_O_2_ concentration and dose of the catechol-HCP-Fe were found to enhance the rate of the reaction. The complete decolouration of 100 ppm MB was observed within 25 min, using 2 M H_2_O_2_ and 2 g/L of catechol-HCP-Fe at room temperature and at a neutral pH. However, considering the reagents’ cost efficiency, the optimised condition for MB degradation was 0.5 M H_2_O_2_ and 1 g/L of catechol-HCP-Fe, which could degrade 100 ppm of MB in around 50 min. The reaction time was discovered to be linearly correlated to the initial dye concentration. The catalyst could also remove MB at high concentrations, up to 500 ppm, within a couple of hours. Catechol-HCP-Fe can be efficiently utilised in wide pH (3–11) and temperature (27–75 °C) ranges. Surprisingly, unlike typical Fenton reactions, the reaction rate increased with an increase in pH. The material can be reused for at least four cycles. Fe can simply be reloaded into the polymer to regenerate the catalyst and improve catalytic efficiency. The plausible reaction mechanism was proposed. The singlet oxygen (^1^O_2_) was presumed to be the dominant reactive species responsible for the MB degradation in the reaction.

This research demonstrated the use of catechol-HCP as an inexpensive material for metal ion nanoconfinement and its application in Fenton catalysis. By using abundant and low-cost chemicals and simple preparation methods, together with its good catalytic property in dye degradation and reusability, catechol-HCP and catechol-HCP-Fe are promising candidates as efficient and inexpensive novel materials for wastewater treatment.

## Figures and Tables

**Table 1:** Elemental analysis of HCPs.

Polymer	% Carbon	% Hydrogen
Catechol-HCP-w	62.68 ± 0.01	3.56 ± 0.12
(Theoretical)	(71.64)	(4.51)
Catechol-HCP-Fe	58.83 ± 0.29	3.44 ± 0.11
(Theoretical) ^1^	(69.85)	(4.40)

^1^ Calculated as 2.5% Fe content (25 mg/g) in the polymer.

**Table 2:** Kinetic constants and *R*^2^ calculated by different kinetic models.

Kinetic Model	Rate Constant (*k*)	Rate Constant Unit	*R* ^2^
Zero-order	2.3175	ppm/min	0.9936
First-order	0.0569	min^−1^	0.9125
Second-order	0.0019	ppm^−1^min^−1^	0.8000

**Table 3:** MB degradation efficiency using different catalysts under visible light.

Material	Dose (g/L)	[MB] (ppm)	[H_2_O_2_] (M)	Efficiency (%)	Time (min)	pH	Reference
FeCl_2_·4H_2_O	0.08	70	0.03	99	120	7	[24]
FeCl_3_·6H_2_O	0.08	70	0.03	99	120	7	[24]
Fe_2_O_3_	0.08	70	0.03	~30	120	7	[24]
Fe_3_O_4_	0.08	70	0.03	~25	120	7	[24]
FePPOP-1	0.08	70	0.03	99	80	7	[24]
FePPOP-1	0.08	100	0.03	99	>120	7	[24]
FcTz-POP	0.2	8	1	99	20	7	[52]
wSF-DA/Fe	0.1	20	1	98 *	10–40 *	7	[58]
Fe_2_O_3_@FCNT-H	0.015	3	0.05	99	60	5	[61]
M-NPs	2	100	0.56	~100	90	3.5	[63]
C-Fe_2_O_3_-2	0.5	50	0.0075	96	420	-	[65]
Fe_3_O_4_@rGO@TiO_2_	1.5	10	0.176	99	120	7	[66]
MIL-53(Fe)	0.01	128	10^−5^	20	20	7	[67]
NTU-9 (Ti(IV)-MOF)	0.5	32	~0.25	99	20–40	7	[68]
Fe, N-CDs	0.5	20	0.147	97 *	60 *	8	[69]
Catechol-HCP-Fe	1	10	0.5	99	21	6	This work
Catechol-HCP-Fe	1	100	0.5	99	51	6	This work
Catechol-HCP-Fe	2	100	2	99	23	6	This work

* Reaction temperature = 50 °C.

## Data Availability

Not applicable.

## Supplementary Materials

The following supporting information can be downloaded at: https://www.mdpi.com/article/10.3390/polym14132749/s1. Figure S1: EDX spectrum and EDX elemental analysis of Catechol-HCP-w; Figure S2: EDX spectrum and EDX elemental analysis of Catechol-HCP-Fe; Figure S3: TGA spectrum of Catechol-HCP-w; Figure S4: TGA spectrum of Catechol-HCP-Fe; Figure S5: Kinetic plots of MB degradation using (a) zero-order, (b) first-order, (c) second-order kinetic model; Figure S6: Degradation efficiency using different initial pH; Figure S7: Degradation efficiency using different reaction temperature; Figure S8: Degradation efficiency of the reused catalyst without Fe reloading; Figure S9: Degradation efficiency of the reused catalyst with Fe reloading; Table S1: EDX elemental analysis (weight%) of HCPs.
