# Supplementary material for: Fe-Immobilised Catechol-Based Hypercrosslinked Polymer as Heterogeneous Fenton Catalyst for Degradation of Methylene Blue in Water
Source: Polymers (Basel). 2022 Jul 5;14(13):2749. doi: 10.3390/polym14132749 (PMC9269043; doi:10.3390/polym14132749)
Supplement: Supplementary file 1 [file polymers-14-02749-s001.zip › polymers-1752615-supplementary.pdf]

Supporting Information

Fe-Immobilised Catechol-Based Hypercrosslinked Polymer as Heterogeneous Fenton Catalyst for Degradation of Methylene Blue in Water

Thanchanok Ratvijitvech [1,*]

[1] Department of Chemistry, Faculty of Science, Mahidol University, Bangkok 10400, Thailand
* Correspondence: thanchanok.rat@mahidol.edu

Table of Figures

Figure S1 . EDX spectrum and EDX elemental analysis of Catechol-HCP-w. 2
Figure S2. EDX spectrum and EDX elemental analysis of Catechol-HCP-Fe. 2
Figure S3. TGA spectrum of Catechol-HCP-w 3
Figure S4. TGA spectrum of Catechol-HCP-Fe. 3
Figure S5. Kinetic plots of MB degradation using (a) zero-order, (b) first-order, (c) second-order kinetic model. 4
Figure S6. Degradation efficiency using different initial pH. 5
Figure S7. Degradation efficiency using different reaction temperature. 5
Figure S8. Degradation efficiency of the reused catalyst without Fe reloading. 6
Figure S9. Degradation efficiency of the reused catalyst with Fe reloading. 7

Table of Tables

Table S1. EDX elemental analysis (weight%) of HCPs. 8

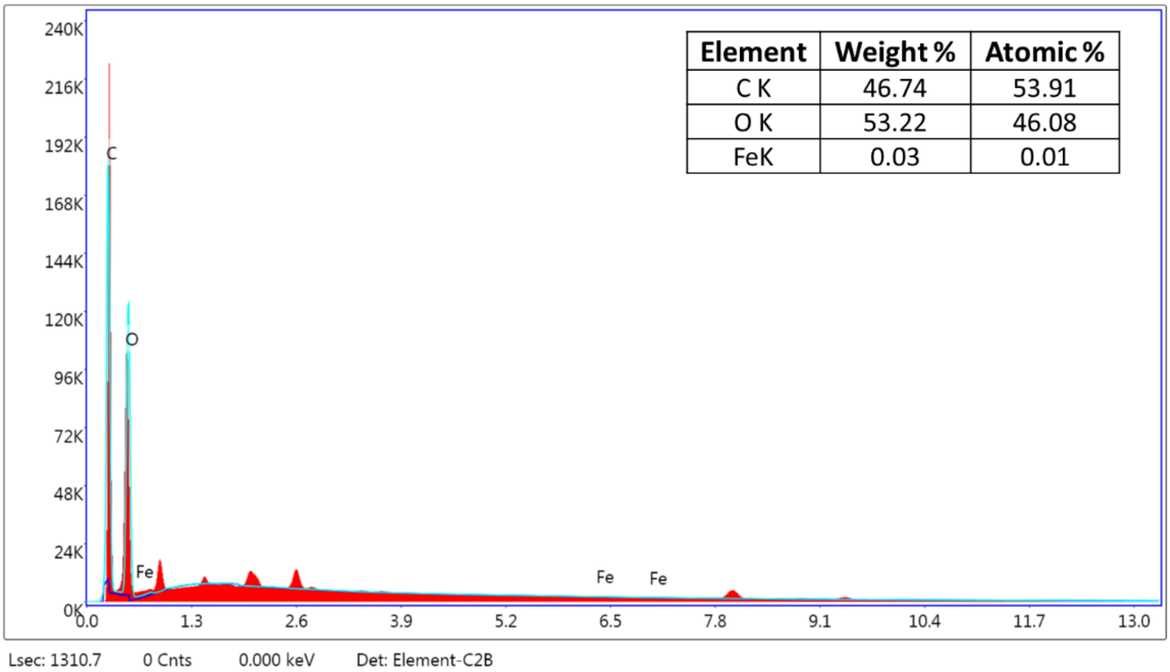

Element	Weight %	Atomic %
C K	46.74	53.91
O K	53.22	46.08
FeK	0.03	0.01

Figure S1 . EDX spectrum and EDX elemental analysis of Catechol-HCP-w.

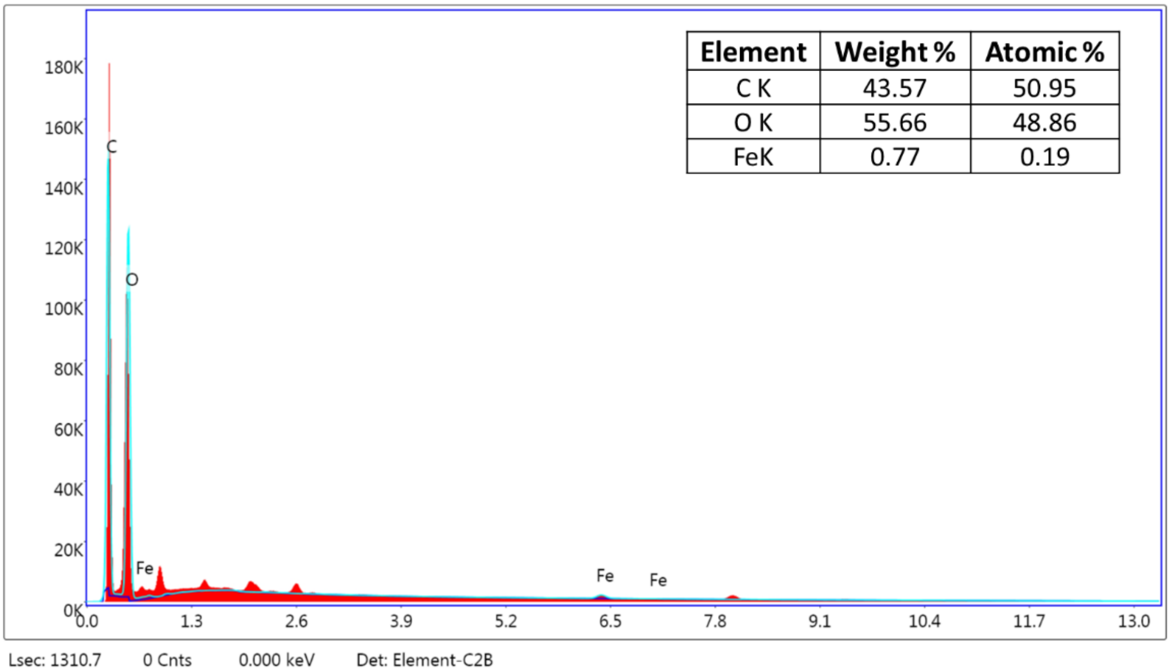

Element	Weight %	Atomic %
C K	43.57	50.95
O K	55.66	48.86
FeK	0.77	0.19

Figure S2. EDX spectrum and EDX elemental analysis of Catechol-HCP-Fe.

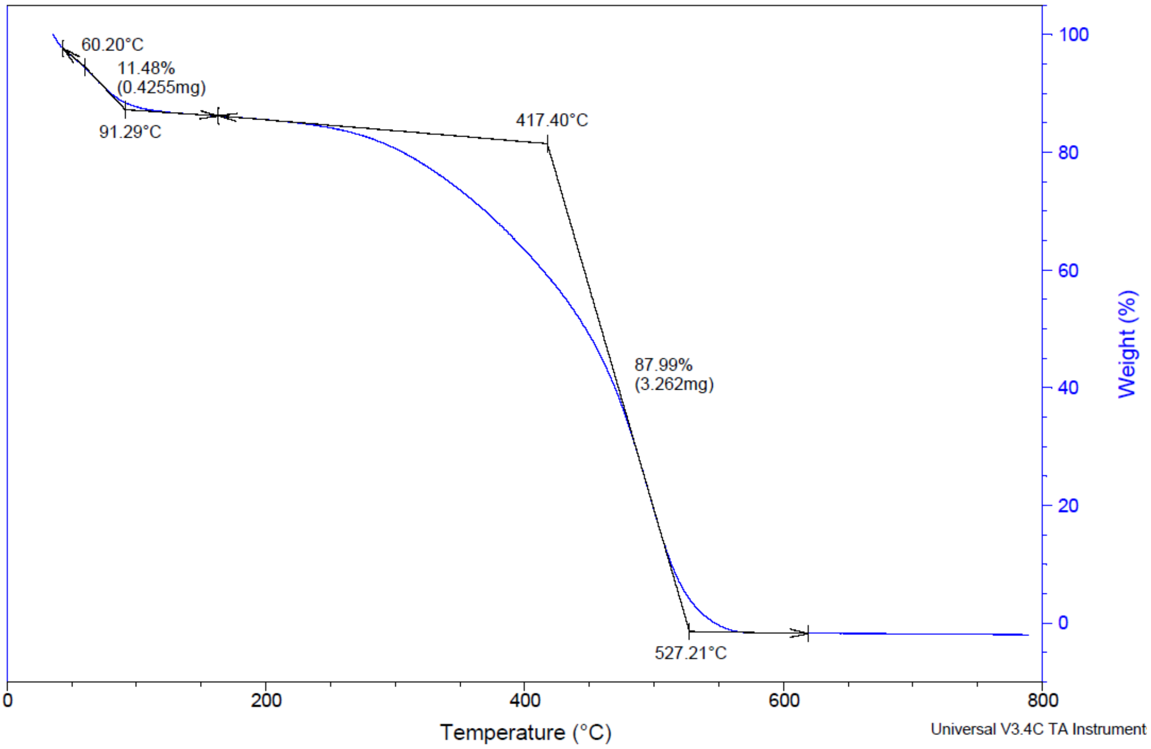

Figure S3. TGA spectrum of Catechol-HCP-w.

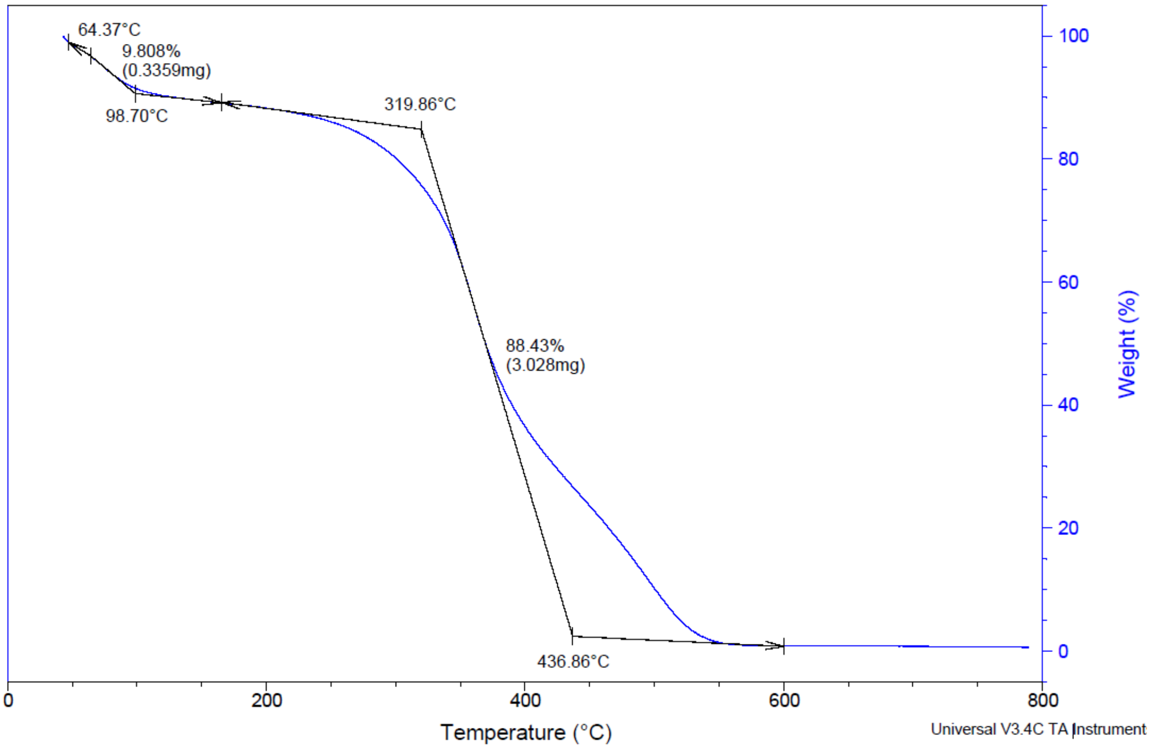

Figure S4. TGA spectrum of Catechol-HCP-Fe.

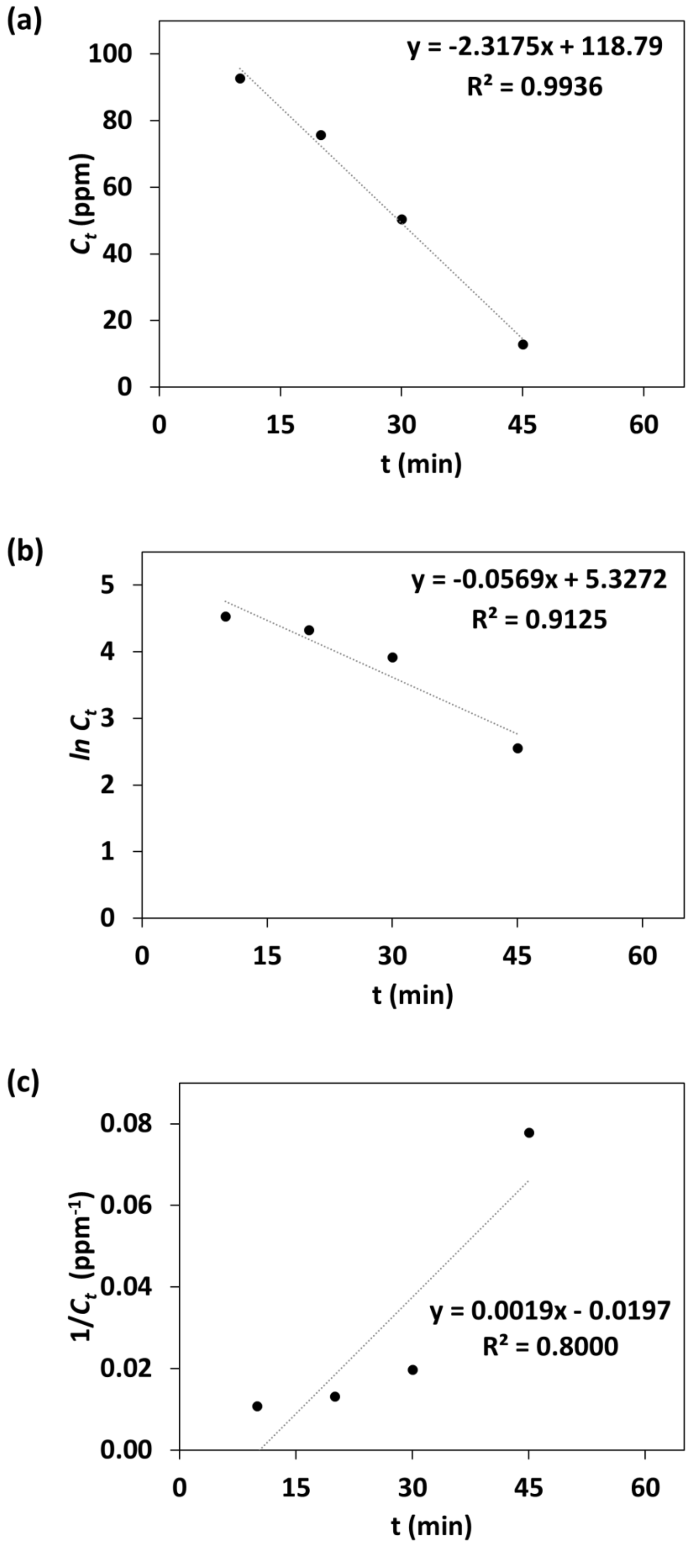

Figure S5. Kinetic plots of MB degradation using (a) zero-order, (b) first-order, (c) second-order kinetic model.

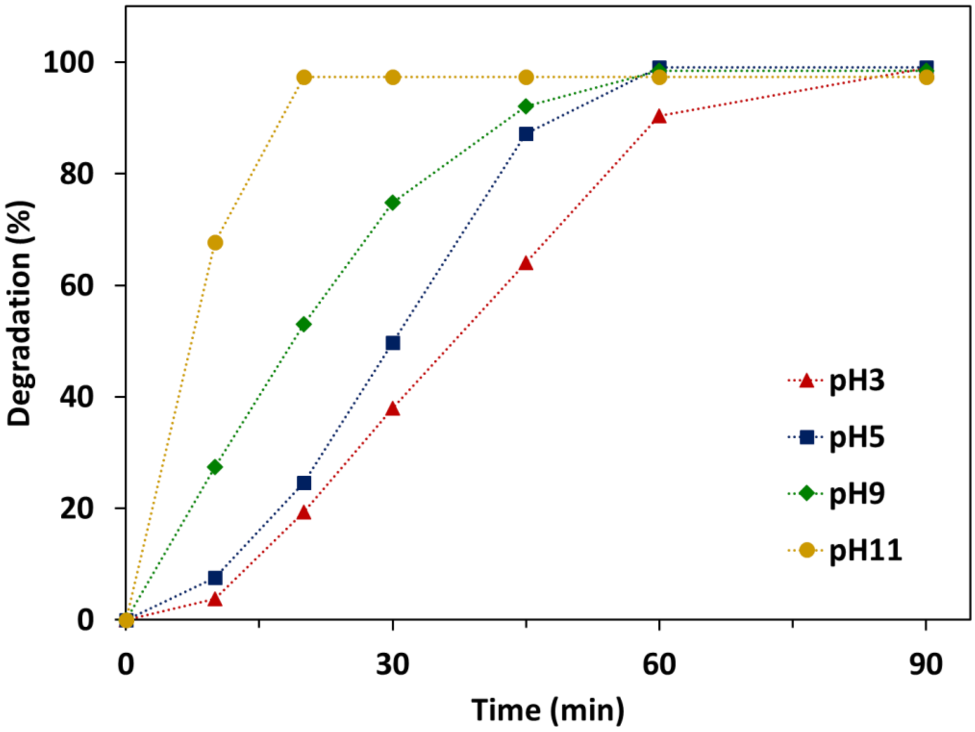

Figure S6. Degradation efficiency using different initial pH.

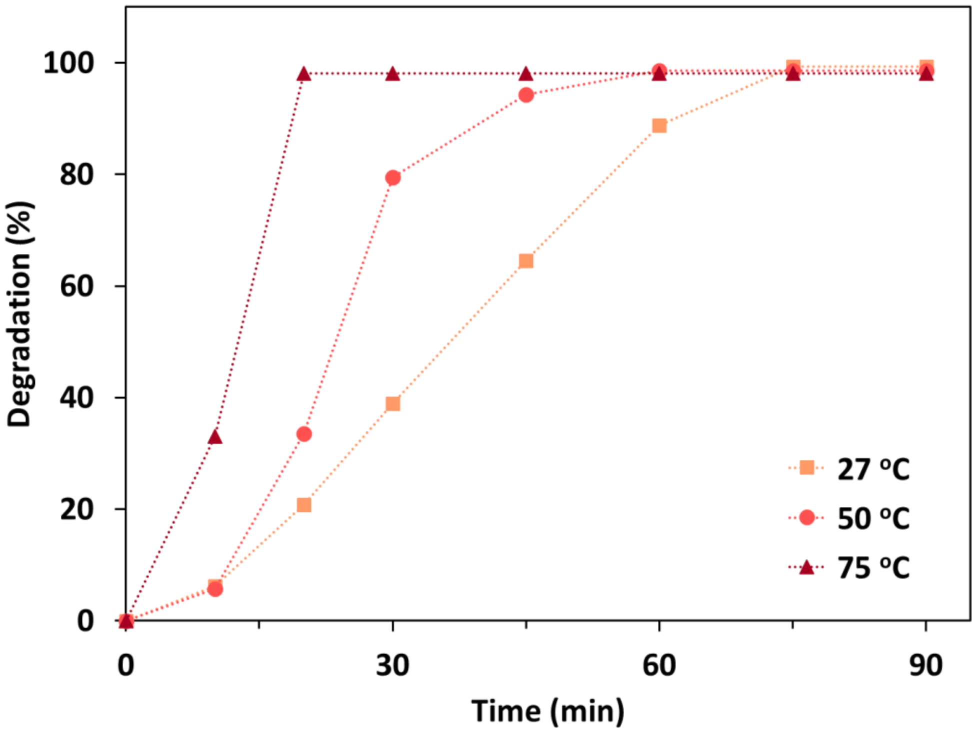

Figure S7. Degradation efficiency using different reaction temperature.

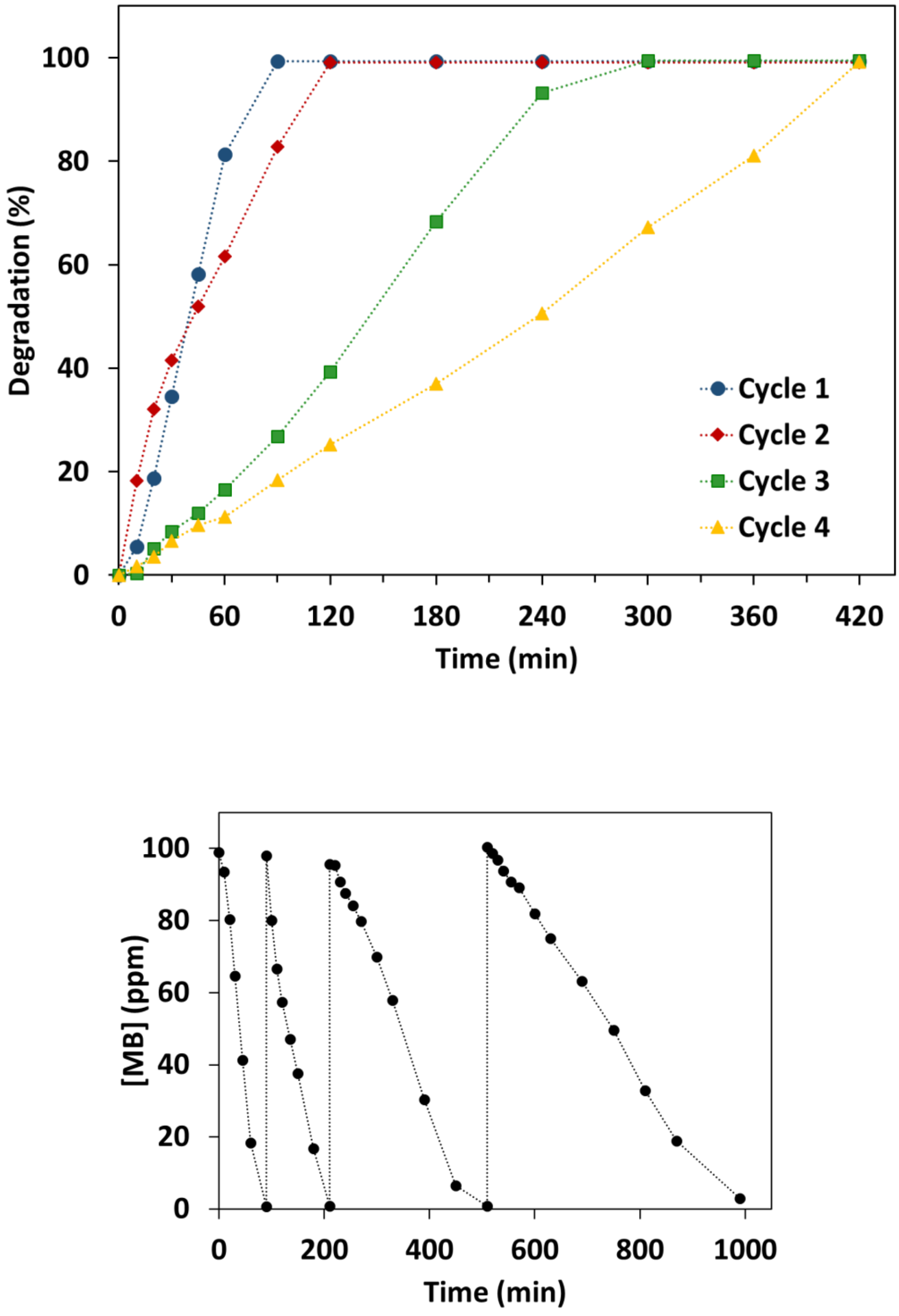

Figure S8. Degradation efficiency of the reused catalyst without Fe reloading.

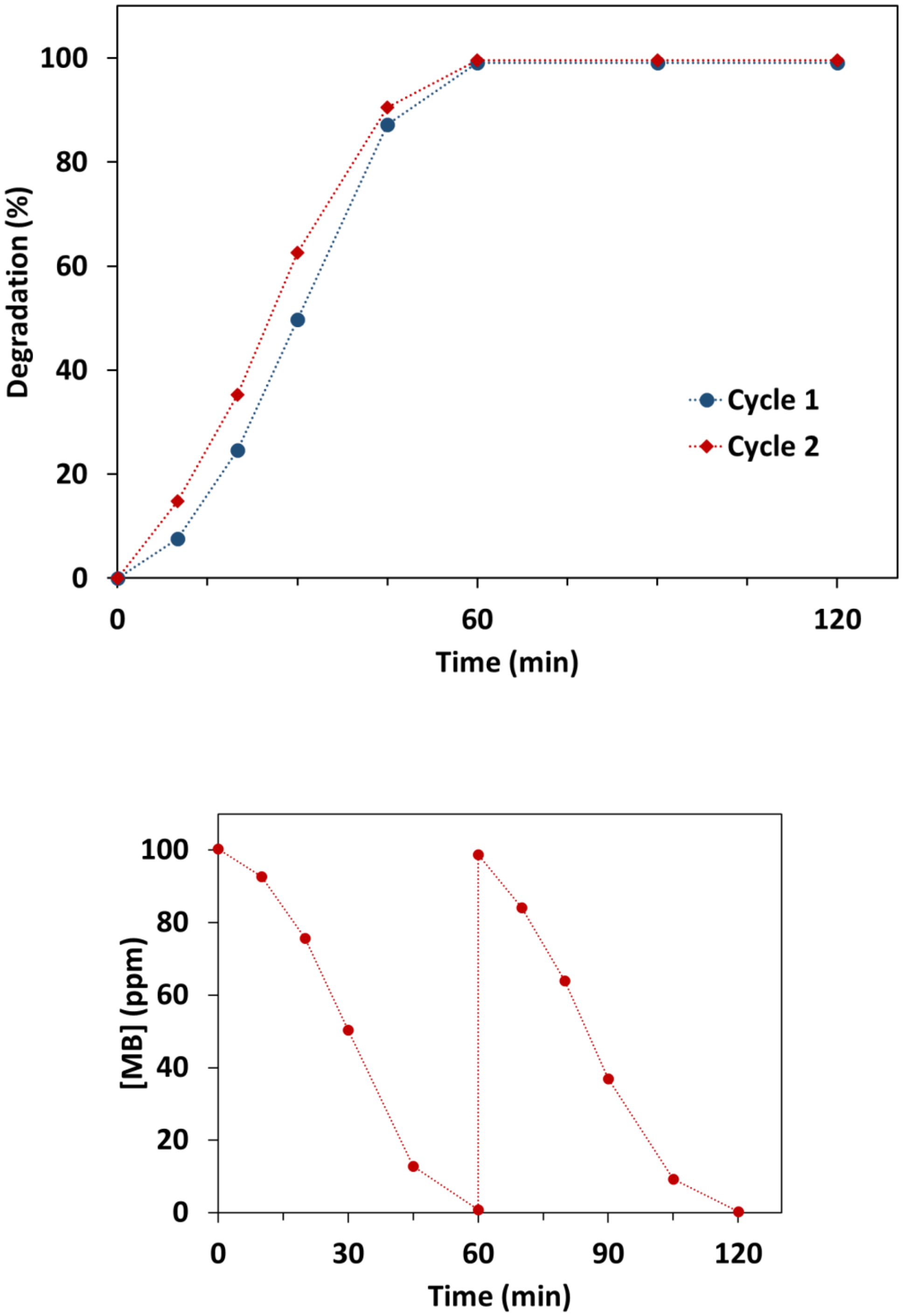

Figure S9. Degradation efficiency of the reused catalyst with Fe reloading.

Table S1. EDX elemental analysis (weight%) of HCPs.

Polymer	%Carbon	%Oxygen	%Iron
Catechol-HCP-w	46.74	53.22	0.03
Catechol-HCP-Fe	43.57	55.66	0.77
